# Endoscopic Ultrasound Elastography for Evaluation of Lymph Nodes: A Single Center Experience

**DOI:** 10.1155/2018/7186341

**Published:** 2018-10-22

**Authors:** Ahmed Youssef Altonbary, Hazem Hakim, Ahmed Mohamed El-Shamy

**Affiliations:** ^1^Department of Gastroenterology and Hepatology, Mansoura Specialized Medical Hospital, Mansoura University, Mansoura, Egypt; ^2^Department of Anesthesia and Intensive Care, Mansoura University, Mansoura, Egypt

## Abstract

**Background and Aim:**

The differentiation between malignant and benign lymph nodes (LNs) is important for tumor staging, for detection of prognosis, and for selection of the best treatment strategy in many cancers. On B-mode EUS, there are some known criteria that suggest the malignant nature of LNs; these criteria may be found in benign LNs. The aim of the work is to evaluate the effectiveness of elasticity score and SR to differentiate between benign and malignant LNs.

**Patients and Methods:**

The study was designed as a retrospective study that included 40 patients with abdominal or mediastinal LNs, either associated with primary masses or isolated, referred for EUS evaluation. Elasticity scores and SR were determined during the examination and finally, EUS-FNA was done at the end of the procedure.

**Results:**

In this 2-years study, 40 patients were enrolled (24 malignant; 16 benign). There were 23 males and 17 females. Their mean age was 52.5 years (range: 28–77). ES alone had a specificity of 87.5%, sensitivity of 41.7%, PPV of 83.3%, NPV of 50%, and accuracy of 60%. Based on the ROC curve analysis results, the best cut-off level of SR to obtain the maximum area under the curve (AUC) was 6.7 with a specificity of 99.9%, sensitivity of 57.1%, PPV of 99.9%, NPV of 64%, and accuracy of 77.5%.

**Conclusion:**

The use of elasticity score and SR increases the reliability of differentiation between benign and malignant LNs and can decrease the number of unnecessary biopsies.

## 1. Introduction

The differentiation between malignant and benign lymph nodes (LNs) is important for tumor staging, for detection of prognosis and for selection of the best treatment strategy in many cancers, as esophageal, stomach, bronchial, and pancreatic tumors. EUS can provide real time images of LNs close to the gastrointestinal tract, but the ability of EUS to differentiate between malignant and benign LNs remains a challenge [1]. On B-mode EUS, there are some known criteria that suggest the malignant nature of LNs (such as hypoechogenicity, rounded shape, sharp borders, and diameter more than 1 cm). However, some of these criteria may be found in benign LNs. Also, it should be considered that none of these typical criteria are found in the early stages of malignant LNs [2]. The accuracy and specificity of these criteria in diagnosis of malignant LNs are considered low [3, 4].

In the past 2 decades, EUS elastography has emerged as a noninvasive tool for estimating the mechanical characteristics of tissues [5]. This technique is used to assess tissue stiffness according to the degree of tissue distortion in response to an external power [6]. The strain ratio (SR), a semiquantitative elastography method, is measured by assessing the elastography pattern of the targeted LN in comparison to that of a nearby reference tissue [7].

In this study, we evaluate the effectiveness of elasticity score and SR to differentiate between benign and malignant LNs.

## 2. Patients and Methods

### 2.1. Patients

Of 309 EUS examinations performed over the 2 years study period from January 2016 to January 2018 at the EUS Unit of the Department of Gastroenterology of Mansoura Specialized Medical Hospital, Mansoura University (Egypt), lymph nodes were detected in 50 patients and 40 of them were included in the study. Eight patients with missing data and two patients with inconclusive biopsy were excluded from the study.

The inclusion criteria were as follows: all patients with abdominal or mediastinal LNs, either associated with primary masses or isolated, referred for EUS evaluation. The exclusion criteria were as follows: patients with a contraindication to interventional endoscopy, as patients with coagulation disorders, or patients unfit for sedation, patients who refused to be involved in the study, and patients in whom the final diagnosis was not known. The study protocol was approved by our ethical committee and written consents were taken from all patients before the procedure.

### 2.2. Methods

The study was designed as a retrospective study to evaluate the effectiveness of elasticity score and SR in diagnosing LNs. On the day of the procedure, eligible patients were appointed to the endoscopy room for EUS examination under intravenous propofol sedation. EUS examination was done in all patients with a Pentax linear Echoendoscope EG3870UTK (PENTAX medical, Tokyo, Japan) attached to a Hitachi Avius ultrasound system (Hitachi Medical Systems, Tokyo, Japan). All EUS examinations were done by two endosonographers. After localization of the LN, elastography was applied to evaluate their hardness. Theoretically, the hardness of malignant LNs is greater than that of inflammatory ones. The hardness of the lesion is assessed by the degree of tissue distortion illustrated on a color map (from red to blue representing soft to hard areas, respectively). Elasticity scores and SR were determined during the examination and finally, EUS-FNA was done at the end of the procedure.

#### 2.2.1. Elasticity Score and Strain Ratio

**Figure 1:**
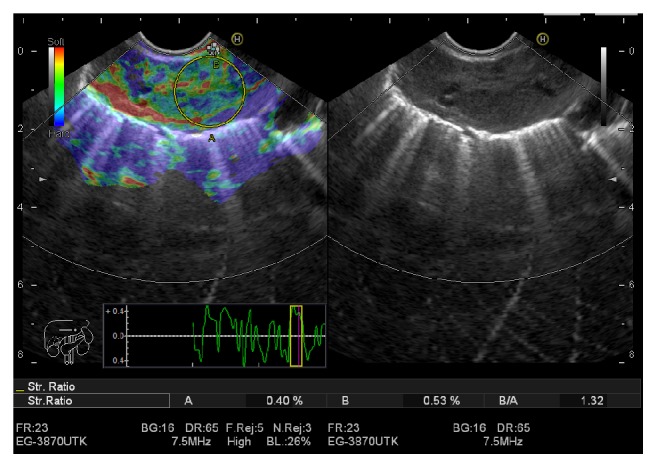
Benign lymph node with elasticity score (ES) 2 and strain ratio (SR) 1.32.

**Figure 2:**
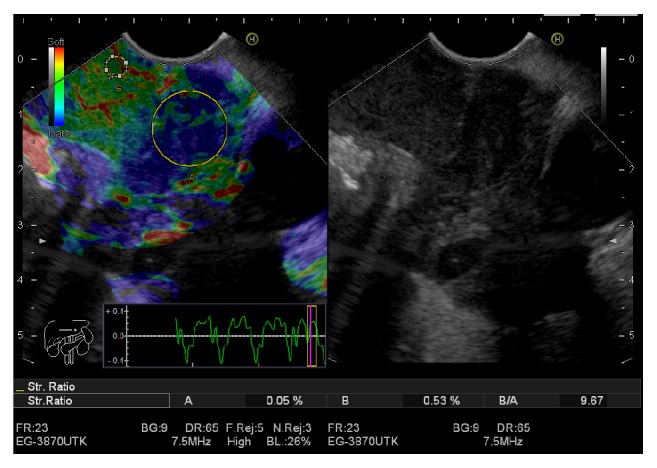
Malignant lymph node with elasticity score (ES) 3 and strain ratio (SR) 9.67.

Elasticity score (ES) was defined as the following: ES 1 was given to homogeneous green and interpreted as normal tissue. ES 2 was given to heterogeneous green predominant and interpreted as inflammation or fibrosis. ES 3 was given to heterogeneous blue predominant and interpreted as indeterminate for malignancy. ES 4 was given to homogeneous blue and interpreted as malignant lesions (Figures 1 and 2).

SR was calculated as the following: two areas were selected, the region of interest selected as area (A) and the normal reference tissue selected as area (B) then dividing area (B) by area (A) (Figures 1 and 2). The final result for SR was calculated from the mean of repeated measures. Subsequently, the receiver operating characteristic (ROC) curve was used to determine the best cut-off value and to calculate the diagnostic value of the SR.

#### 2.2.2. Statistical Analysis

IBM SPSS statistics for widows (version 24) was used for calculating the means of strain ratios, sensitivity, specificity, positive predictive value (PPV), negative predictive value (NPV), and accuracy. The best cut-off value was selected by comparing diagnosis made by elasticity score, SR, and final diagnosis obtained by the cytopathological examination of the EUS-FNA samples or after surgical excision using the receiver operating characteristic (ROC) curve and was used to calculate the diagnostic value. Shapiro-Wilk test was used to check the normality of data and Student t-test and Mann-Whitney Tests were used for parametric and nonparametric data, respectively, with 95% confidence interval (CI). P value ≤ 0.05 was considered statistically significant.

## 3. Results

In this 2-year study, 40 patients were enrolled (24 malignant, 16 benign). There were 23 males and 17 females. Their mean age was 52.5 years (range: 28-77). The size, site, final diagnosis of lymph nodes, SR, and elasticity score are shown in Tables 1–5.

ES 1 and 2 were deemed benign while ES 3 and 4 were deemed malignant. ES alone had a specificity of 87.5%, sensitivity of 41.7%, PPV of 83.3%, NPV of 50%, and accuracy of 60% (Table 6).

There was a significant statistical difference between the mean value of the SR for benign LNs (3) and the mean value of the SR for malignant LNs (17) (p=0.001). Based on the ROC curve analysis results, the best cut-off level of SR to obtain the maximum area under the curve (AUC) was 6.7 with a specificity of 99.9%, sensitivity of 57.1%, PPV of 99.9%, NPV of 64%, and accuracy of 77.5% (Table 6).

## 4. Discussion

The present study provides evidence supporting EUS elastography as an accurate and useful tool for the differential diagnosis of LNs. Strain ratio (SR) adds important and objective information to EUS by providing a quantitative evaluation of tissue stiffness, which supports the benign or malignant nature of LNs.

Although EUS-FNA is considered as the gold standard for the diagnosis of malignant LNs, with PPV and specificity approaching 100%, FNA needs appropriate training and good experience and may be associated with many complications [8]. Recently, many studies showed that EUS elastography and SR are useful tools in assessing LNs and selecting the suspected nodes. Also, EUS elastography can be used to target the hardest area in the LN which is considered the most suspicious area of the node before tissue sampling. In case of multiple LNs, EUS elastography can increase the sensitivity of EUS-FNA by reducing the number of unnecessary biopsies. Moreover, in patients with negative EUS-FNA or in situations in which FNA cannot be done (technical difficulties or interposed blood vessel), EUS elastography can be used as a useful alternative to differentiate malignant from benign LNs [9, 10].

Okasha and his colleges evaluated the value of the SR in the differentiation between benign and malignant LNs. They examined 126 LNs, with a SR cut-off value of 4.6 for malignant LNs. The specificity, sensitivity, NPV, and PPV were 83.3%, 89.8%, 90.2%, and 82.5%, respectively [11].

Paterson and his colleges evaluated the value of the SR in the LN staging of esophageal and gastric tumors, using EUS-FNA cytology as the reference method. They examined 50 LNs, with a SR cut-off value of 7.5 for malignant LNs. The specificity, sensitivity, NPV, PPV, and accuracy were 96%, 83%, 86%, 95%, and 90%, respectively, compared to the values of 22–70%, 64–96%, 61–83%, 57–72%, and 60–75%, respectively, that were gained from different criteria of B-mode EUS [12].

Larsen and his colleges assessed the use of EUS elastography and SR in the evaluation of nodes present with upper gastrointestinal tract malignancies, using surgical pathology as the reference method. A total of 56 LNs were evaluated. For EUS elastography, the specificity, sensitivity, PPV, NPV, and accuracy were 85%, 55%, 71%, 74%, and 73%, respectively. And for the SR at a cut-off value of 4.5, the specificity, sensitivity, PPV, NPV, and accuracy were 82%, 55%, 67%, 74%, and 71%, respectively [13].

In our study, ES alone had a specificity of 87.5%, sensitivity of 41.7%, PPV of 83.3%, NPV of 50%, and accuracy of 60%. This was in contrast to the previous published study by Larsen et al. [13] that showed higher sensitivity, NPV, and accuracy. This may be attributed to the subjectivity of ES.

Many studies mentioned the SR with different cut-off levels to overcome the subjectivity and increase specificity of EUS elastography. We had a cut-off level of 6.7 that had specificity, sensitivity, PPV, NPV, and accuracy of 99.9%, 57%, 99.9%, 64%, and 77.5%, respectively. This was comparable to the study done by Paterson et al. [12] that identified a cut-off level of 7.5 that had specificity and PPV of 96% and 95%, respectively, but higher values of sensitivity, NPV, and accuracy of 83%, 86%, and 90%, respectively. Okasha et al. [11] and Larsen et al. [13] identified a lower cut-off value for SR of 4.6 and 4.5 that gave lower values in specificity and PPV of 83.3%, 82.5% and 82%, 67%, respectively.

The present study has some limitations as follows: EUS elastography and SR were used to evaluate different types of pathology in malignant nodes (e.g., mediastinal, pancreatic, perigastric, paraaortic, etc.) and benign nodes, which may cause divergence in the results due to different tissue heterogeneity. Also, relatively small number of patients are compared to other studies. On the other hand, the use of elasticity score and strain ratio may be useful alternative, in patients with negative EUS-FNA or in situations in which FNA cannot be done.

In conclusion, the use of elasticity score and SR increases the reliability of differentiation between benign and malignant LNs and can decrease the number of unnecessary biopsies.

## Figures and Tables

**Table 1:** Location of the lymph nodes.

**Location**	**Number of cases =40**	
	**Frequency**	**Percent**
Mediastinum	16	40%

Portahepatis	8	20%

Para-aortic	8	20%

Perigastric	5	12.5%

Perirectal	2	5%

Paraesophageal	1	2.5%

**Table 2:** Size (cm) of the lymph nodes (n = 40).

	**All lesions** **(n= 40)**	**Benign lesions** **(n= 16)**	**Malignant lesions** **(n= 24)**	**P value**
Mean± SD	4.3±1.7	3.7±1.3	4.9±1.7	0.021

Median	4.0	3.5	5	

Range	1.1: 9.0	2.0: 6.0	1.1: 9.0	

**Table 3:** Diagnosis of the lymph nodes (n = 40).

**Diagnosis**		**Frequency**	**Percent**
**Benign**	Inflammatory	14	35%
	Thymoma	1	2.5%
	Sarcoidosis	1	2.5%

**Malignant**	Lymphoma	13	32.5%
	Undifferentiated carcinoma	7	17.5%
	Adenocarcinoma	3	7.5%
	Small cell carcinoma	1	2.5%

**Table 4:** Strain ratio of the lymph nodes.

**Elastography**	**All lesions** **(n= 40)**	**Benign lesions** **(n= 16)**	**Malignant lesions** **(n= 24)**	**P value**
Mean± SD	10.9±23.5	3±1.1	17±30	0.001

Median	3.4	2.7	9.5	

Range	1.7: 140	1.7: 5.4	2: 140	

**Table 5:** Elasticity score of the lymph nodes.

	**All lesions** **(n= 40)**		**Benign lesions** **(n= 16)**		**Malignant lesions** **(n= 24)**	
	**Frequency**	**Percent**	**Frequency**	**Percent**	**Frequency**	**Percent**
**ES 2**	28	70%	14	87.5%	14	58.3%

**ES 3**	12	30%	2	12.5%	10	41.7%

**Table 6:** Diagnostic values of ES and SR of the lymph nodes.

	**ES**	**SR 6.7**
Sensitivity	41.7%	57.1%

Specificity	87.5%	99.9%

PPV	83.3%	99.9%

NPV	50%	64%

Accuracy	60%	77.5%

## Data Availability

The data supporting this study are from previously reported studies, which have been cited in the article. SPSS sheet also has been included in the email and the data supporting this study are available from author upon request.

## Supplementary Materials

Supplementary Materials Supplementary file is SPSS sheet including description of demographic data, elastography, strain ratio, and final diagnosis of all patients.
