# Supplementary material for: Endoscopic Ultrasound Elastography for Evaluation of Lymph Nodes: A Single Center Experience
Source: Diagn Ther Endosc. 2018 Oct 22;2018:7186341. doi: 10.1155/2018/7186341 (PMC6217875; doi:10.1155/2018/7186341)
Supplement: Supplementary Materials — Supplementary file is SPSS sheet including description of demographic data, elastography, strain ratio, and final diagnosis of all patients. [file 7186341.f1.pdf]

ID	Age	Sex	Site	Nature	Size	EUS_E	EUS_E	Nature.p	Diagnosis
1	65.00	male	mediastinum	solid lesion	5.50	ES 2		Malignant	lymphoma
2	44.00	male	paraoartic	solid lesion	6.50	ES 2		Malignant	undiff carcinoma
3	59.00	female	paraoartic	solid lesion	5.00	ES 2		Malignant	lymphoma
4	47.00	female	mediastinum	solid lesion	5.60	ES 2	1.70	Benign	sarciodosis
5	49.00	male	perirectal	solid lesion	2.00	ES 2	1.80	Benign	inflammatory
6	47.00	female	mediastinum	solid lesion	3.50	ES 2	1.90	Benign	inflammatory
7	46.00	female	mediastinum	solid lesion	4.00	ES 2	1.90	Benign	inflammatory
8	38.00	female	paraoartic	solid lesion	3.50	ES 2	2.00	Malignant	lymphoma
9	77.00	male	mediastinum	solid lesion	1.10	ES 2	2.10	Malignant	small cell carcinoma
10	35.00	male	mediastinum	solid lesion	5.50	ES 2	2.20	Benign	thymoma
11	66.00	male	mediastinum	solid lesion	2.50	ES 2	2.40	Benign	inflammatory
12	53.00	female	paraoartic	solid lesion	4.50	ES 2	2.40	Benign	inflammatory
13	52.00	male	mediastinum	solid lesion	4.00	ES 2	2.40	Malignant	lymphoma
14	40.00	male	portahepatis	solid lesion	2.00	ES 2	2.60	Benign	inflammatory
15	47.00	male	perigastric	solid lesion	4.60	ES 2	2.70	Benign	inflammatory
16	62.00	male	mediastinum	solid lesion	9.00	ES 2	2.80	Malignant	lymphoma
17	63.00	male	paraoartic	solid lesion	6.60	ES 2	2.80	Malignant	lymphoma
18	52.00	male	mediastinum	solid lesion	4.00	ES 2	3.00	Malignant	lymphoma
19	64.00	female	perirectal	solid lesion	3.00	ES 3	3.00	Benign	inflammatory
20	51.00	male	paraesophageal	solid lesion	3.50	ES 2	3.10	Benign	inflammatory
21	40.00	female	portahepatis	solid lesion	6.00	ES 2	3.40	Benign	inflammatory
22	41.00	male	paraoartic	solid lesion	4.00	ES 2	3.40	Malignant	lymphoma
23	67.00	female	mediastinum	solid lesion	3.40	ES 3	3.80	Benign	inflammatory
24	53.00	male	portahepatis	solid lesion	2.00	ES 2	4.50	Benign	inflammatory
25	40.00	female	mediastinum	solid lesion	3.50	ES 2	4.50	Benign	inflammatory
26	58.00	male	mediastinum	solid lesion	6.00	ES 2	4.50	Malignant	undiff carcinoma
27	63.00	male	mediastinum	solid lesion	3.00	ES 2	4.80	Malignant	lymphoma
28	38.00	female	portahepatis	solid lesion	3.00	ES 2	5.40	Benign	inflammatory
29	38.00	male	portahepatis	solid lesion	2.00	ES 2	8.00	Malignant	lymphoma
30	59.00	male	mediastinum	solid lesion	6.00	ES 3	9.50	Malignant	undiff carcinoma
31	68.00	female	portahepatis	solid lesion	3.90	ES 3	9.50	Malignant	undiff carcinoma
32	64.00	female	perigastric	solid lesion	3.00	ES 3	11.50	Malignant	adenocarcinoma
33	62.00	female	mediastinum	solid lesion	5.50	ES 3	12.00	Malignant	undiff carcinoma

34	49.00	female	portahepatis	solid lesion	4.50	ES 3	13.00	Malignant	lymphoma
35	58.00	female	paraoartic	solid lesion	7.00	ES 3	19.50	Malignant	lymphoma
36	64.00	male	perigastric	solid lesion	6.00	ES 2	20.00	Malignant	adenocarcinoma
37	28.00	male	perigastric	solid lesion	5.50	ES 3	20.00	Malignant	adenocarcinoma
38	53.00	male	perigastric	solid lesion	5.00	ES 3	23.50	Malignant	undiff carcinoma
39	37.00	male	paraoartic	solid lesion	3.80	ES 3	45.00	Malignant	lymphoma
40	58.00	female	portahepatis	solid lesion	6.00	ES 3		Malignant	undiff carcinoma
